# Implementation of Non-Linear Non-Parametric Persistent Scatterer Interferometry and Its Robustness for Displacement Monitoring

**DOI:** 10.3390/s21031004

**Published:** 2021-02-02

**Authors:** Fumitaka Ogushi, Masashi Matsuoka, Marco Defilippi, Paolo Pasquali

**Affiliations:** 1 Department of Built Environment, Tokyo Institute of Technology, Yokohama 226-8502, Japan; 2 Department of Architecture and Building Engineering, Tokyo Institute of Technology, Yokohama 226-8502, Japan; matsuoka.m.ab@m.titech.ac.jp; 3 Sarmap S.A., 6987 Caslano, Switzerland; mdefilippi@sarmap.ch (M.D.); ppasquali@sarmap.ch (P.P.)

**Keywords:** SAR, interferometric stacking, persistent scatterer, multi-baseline, scattering distribution, EV spectrum

## Abstract

To derive surface displacement, interferometric stacking with synthetic aperture radar (SAR) data is commonly used, and this technique is now in the implementation phase in the real world. Persistent scatterer interferometry (PSI) is one of the most universal approaches among in- terferometric stacking techniques, and non-linear non-parametric PSI (NN-PSI) was proposed to overcome the drawbacks of PSI approaches. The estimation of the non-linear displacements was successfully conducted using NN-PSI. However, the estimation of NN-PSI is not always stable with certain displacements because wider range of the velocity spectrum is used in NN-PSI than the conventional approaches; therefore, a calculation procedure and parameter optimization are needed to consider. In this paper, optimized parameters and procedures of NN-PSI are proposed, and real data processing with Sentinel-1 in the Kanto region in Japan was conducted. We confirmed that the displacement estimation was comparable to the measurement of the permanent global positioning system (GPS) stations, and the root mean square error between the GPS measurement and NN-PSI estimation was less than 3 mm in two years. The displacement over 2π ambiguity, which the conventional PSI approach wrongly reconstructed, was also quantitatively validated and successfully estimated by NN-PSI. As a result of the real data processing, periodical displacements were also reconstructed through NN-PSI. We concluded that the NN-PSI approach with the proposed parameters and method enabled the estimation of several types of surface displacements that conventional PSI approaches could not reconstruct.

## 1. Introduction

In the past decade, the number of operative spaceborne synthetic aperture radar (SAR) sensors has increased dramatically. In this circumstance, the use of multi-temporal images is essential, and one of the techniques to monitor surface displacements with SAR data processing is called interferometric stacking [1]. Among the various interferometric stacking techniques persistent scatterer interferometry (PSI) [2] has become one of the most common approaches that enable one to measure millimetric order surface displacements. A great deal of research has been performed using these approaches to detect surface displacements of infrastructures such as buildings, railways, highways, paved roads, dams, and other artificial objects [3,4,5,6,7,8,9,10,11,12]. There are also many studies that extended the PSI technique to improve the accuracy of the displacement estimation and to solve certain limitations [13,14,15,16,17]. One of the significant limitations in the PSI technique is in estimating non-linear displacements. The estimation for displacements in PSI is based on a linear model, and the displacements are often wrongly estimated when the actual displacement is non-linear. To estimate the non-linear displacement using PSI approaches, it is common to use quite complicated models and empirical data for the region of interest [18,19,20], or assume some kind of spatial correlation of the displacement to be estimated [21]. 

In our previous study, we proposed an extended PSI method, called non-parametric non-linear PSI (NN-PSI) [22], and the non-linear displacement, which the conventional PSI (ConvPSI) wrongly estimates, was appropriately estimated. NN-PSI is supposed to estimate many types of the displacements by using the complete velocity spectrum in the elevation-velocity (EV) spectrum, whereas ConvPSI only uses single frequency when reconstructing displacement phase. The reconstruction of NN-PSI is quite flexible and powerful, and no non-linear displacement models and empirical parameters are used. One of the most significant advantages is that the whole estimation is conducted within a single pixel. This means that the spatial filters and unwrapping that usually use adjacent pixels are not implemented in NN-PSI. The critical part in NN-PSI is to exploit an EV spectrum that shows correlations between the residual height and velocity phase, so that it is possible to obtain the optimized parameters of residual height and velocity, and eventually the best residual height, which is used in the displacement reconstruction, can be selected. The details of NN-PSI and EV spectrum exploitation are explained as background information in Section 2.

In [22], the subsidence influenced by the subway construction in a short period in Budapest was successfully estimated by NN-PSI. However, only one type of non-linear displacement was validated, and it is necessary to examine if NN-PSI is also applicable to other types of non-linear displacements. In addition to estimating other types of displacements, the procedure to exploit the EV spectrum in NN-PSI should be generalized because the algorithm is simple in estimating the non-linear displacement; however, it is also easy to obtain erroneous results. Since a wider range of the spectrum [23] is used in NN-PSI than ConvPSI, the resulting displacement of NN-PSI is more influenced by the phase noise than that of ConvPSI. It is critical for NN-PSI to standardize the exploitation of the EV spectrum. Concretely the way to decide the calculation range of the EV spectrum and the height must be investigated.

Therefore, in this paper, the calculation range for EV spectrum in the NN-PSI procedures is discussed, based on the multi-baseline model, and a robust method to detect the height for the displacement estimation is proposed by using a spectral analysis in the EV spectrum. The proposed method was verified with several types of non-linear displacements using simulations. Furthermore, the proposed method was applied to Sentinel-1 data as a validation of the real data processing.

**Figure 1:**
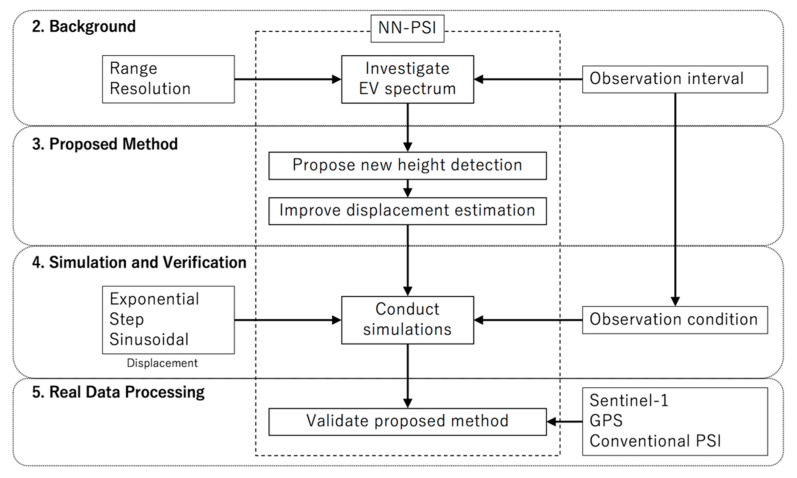
The flowchart of the sections in this paper.

The remainder of this paper is structured as shown in Figure 1. In Section 2, the basic concept of the NN-PSI and multi-baseline approach, such as the generation of EV spectrum, and the importance of observation intervals are reviewed. In Section 3, a method to extract an appropriate height for the displacement estimation is proposed. In Section 4, three types of displacements are investigated by simulation with the proposed method, and also the effect of the observation intervals is investigated. In Section 5, an experiment with actual observation data processing is presented. In Section 6, the results in the previous sections are discussed, and the conclusions are presented in the last section.

## 2. Background

### 2.1. Review of NN-PSI

**Figure 2:**
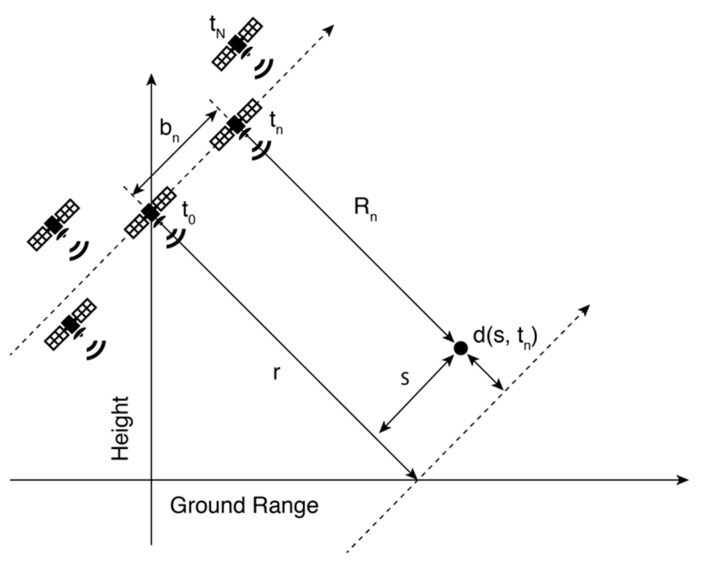
System geometry in the plane orthogonal to the orbit direction. Each satellite shows the positions of the acquisition antennas over repeated passes [25].

The NN-PSI approach was proposed in [22], and the main concept is summarized in this subsection. NN-PSI is based on the single-master and multi-baseline model [23,24,25,26,27], illustrated in Figure 2. Each received signal can be derived from the range distance *R_n_* in any pixel at the *n*^th^ acquisition that is expressed by the baseline distance *b_n_*, the reference range distance *r*, the elevation direction *s*, and the displacement in the line of sight *d(s, t_n_)*.

Then, a received complex signal $g_{n}$ is composed of a 2-D Fourier transform of the scattering distribution in the EV spectrum in Equation (1):

(1) $$g_{n}=\overset{s_{max}}{\underset{s_{min}}{\int }}\overset{v_{max}}{\underset{v_{min}}{\int }}a_{\gamma }\left(s,v\right)\exp\left(j2\pi (\xi_{n}s+\eta_{n}v\right)dvds$$

where $s_{min}$ and $s_{max}$ are the range of height, $v_{min}$ and $v_{max}$ are the range of velocity, $a_{\gamma }\left(s,v\right)$ is the scattering distribution, $\xi_{n}=2b_{n}/\left(\lambda r\right)$ are the sampling spatial frequencies, and $\eta_{n}=2t_{n}/\lambda$ are the temporal frequencies. The equation indicates that the displacement can be reconstructed when the scattering distribution is correctly derived by the given range of height and velocity. In the NN-PSI approach, the scattering distribution is estimated by the temporal coherence [26], so that the best combination of the velocity and height can be selected according to the temporal coherence. The height for the displacement estimation *s_0_* can be selected using the statistics of the temporal coherence in the given EV spectrum. Once the height is decided, the scattering distribution is turned into the spectral distribution $\gamma_{t}\left(s_{0},v\right)$, which is the profile of the temporal coherence at the selected height. The displacement is reconstructed by Equation (2):

(2) $$\exp\left(j4\pi d\left(s_{0},t_{n}\right)/\lambda \right)=\overset{v_{max}}{\underset{v_{min}}{\int }}\gamma_{t}\left(s_{0},v\right)\exp(j2\pi (\xi_{n}s_{0}+\eta_{n}v))dv$$

According to Equations (1) and (2), the resulting displacements of NN-PSI are heavily dependent on the ranges of the velocity and selected height for the displacement estimation. In the previous study, the ranges of these parameters were arbitrarily decided, and the way to select the height with the maximum coherence in the EV spectrum did not always lead to the correct displacement estimation with certain non-linear displacements. Therefore, the important parameters to generate the EV spectrum are discussed in the next subsections.

### 2.2. The Range and Resolution of EV Spectrum

As explained in Introduction, the height and velocity ranges, which are the size of the EV spectrum, impact the height selection, and this can be explained by Equation (1). The size of the EV spectrum can be defined based on the height and velocity ambiguity [27]. Since the measurement of the height and displacement with SAR is limited to the half of its wavelength, 2π, the multi-baseline model also follows these ambiguities. The height and velocity ambiguities, $\Delta_{s}$ and $\Delta_{v}$, can be calculated using Equations (3) and (4), respectively:

(3) $$\Delta_{s}=\lambda r/2\Delta b$$

(4) $$\Delta_{v}=\lambda /2\Delta t$$

where λ is the wavelength of the SAR sensor, r is the slant range distance, Δb is the average of the spatial baseline distance, and Δt is the average temporal baseline of the interferometric pairs. Theoretically, the signal information is periodic if the calculation range exceeds the ambiguity, and the maximum range of the EV spectrum can be defined by the height and velocity ambiguity.

Regarding the size of the EV spectrum, the height and velocity resolutions [14,23] are also important parameters to consider. These parameters can be derived using Equations (5) and (6):

(5) $$\delta_{s}=\lambda r/\left(2B\right)$$

(6) $$\delta_{v}=\lambda /\left(2T\right)$$

where *B* is the total spatial baseline in the observation period, and *T* is the total observation period. These resolutions are the maximum resolutions to estimate the temporal coherence at the given height and velocity in the EV spectrum.

In summary, the ranges of the EV spectrum in the height and velocity directions are defined by the height and velocity ambiguity,$\;\Delta_{s}$ and $\Delta_{v}$. The resolution of the height and velocity in the EV spectrum is defined by the height and velocity resolution, $\delta_{s}$ and $\delta_{v}$. In principle, the maximum ranges in the EV spectrum are decided by $\Delta_{s}$ and $\Delta_{v}$, and sampling sizes for the EV spectrum can be decided by $\delta_{s}$ and $\delta_{v}$. We noted that the oversampling of these resolutions is recommended. In our preliminary experiment, the smaller sampling sizes provided better displacement estimations with NN-PSI. Therefore, the height and velocity sampling sizes can be set with smaller numbers compared to the values of $\delta_{s}$ and $\delta_{v}$.

**Figure 3:**
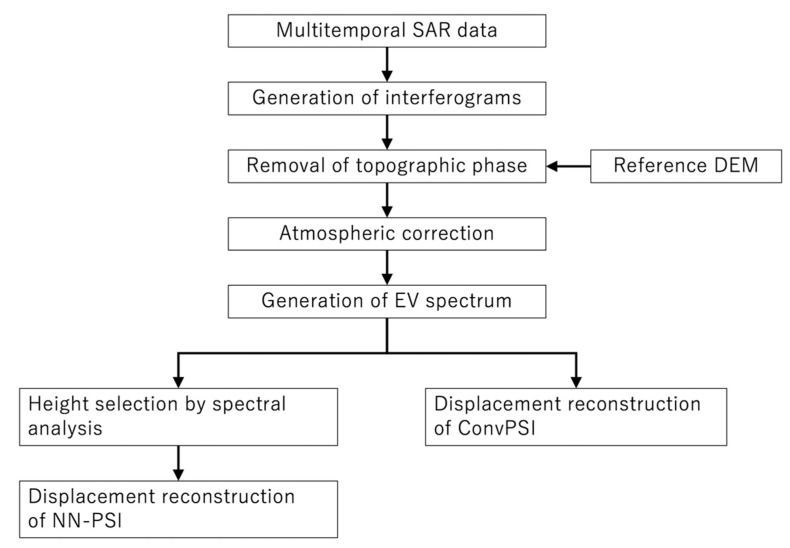
The flowchart shows the whole data processing of NN-PSI and ConvPSI.

In order to make clear the whole data process for NN-PSI, the workflow is shown in Figure 3. In the simulation and real data processing explained in the later sections, ConvPSI was also conducted in order to compare the results, and the data process of the ConvPSI is also shown in the figure.

### 2.3. Observation Intervals

**Figure 4:**
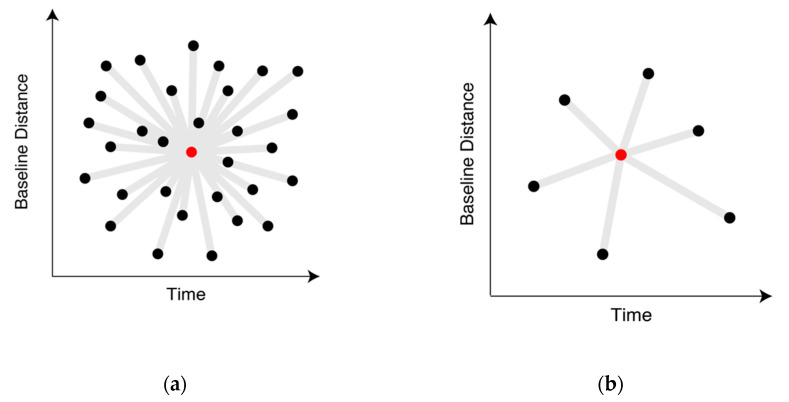
The distribution of spatial baselines related with the acquisition times. (**a**) shows dense acquisitions for multi-baseline model. (**b**) shows sparse baselines and acquisitions [27]. The red point shows the reference observation in the multi-baseline model, the black point shows other observations, and the gray line shows the connections of the pairs.

NN-PSI based on the multi-baseline model is required to use many spatial baselines and acquisition times [27], illustrated in Figure 4a, in order to conduct spectral analysis with the EV spectrum; however, in the practical situation, depicted in Figure 4b, the spaceborne satellite is mostly operated with single baseline and irregular observation intervals. This often leads to unsatisfactory ranges and resolutions of EV spectrum to reconstruct the displacement correctly. Therefore, the impact of the sparse acquisitions should be investigated as a limitation of NN-PSI.

The recent satellite observations are operated with small baseline distances and the variation of the spatial baseline is not so large. Thus, the impact of the sparse observation intervals, which are equivalent to temporal baselines, is mainly investigated rather than that of spatial baselines in this paper.

## 3. Proposed Method

In our previous study [22], the height *s_0_* for the displacement estimation was decided by the maximum temporal coherence in the given EV spectrum. This method works properly for many types of non-linear displacements. However, some periodical displacements cannot be reconstructed due to the wrong height selection. With the periodical displacements, the maximum temporal coherence in the EV spectrum does not always show the height that should be used for the displacement estimation. Instead of searching for the maximum coherence for the correct height detection, the following method is proposed:

(1) Decide the range and sampling resolution of EV spectrum by the ambiguity based on the observation conditions.
(2) Select the height by the minimum average value of the total coherence values at each height in the velocity direction.
(3) Extract coherence profile along the velocity direction at the selected height, $\gamma_{t}\left(s_{0},v\right)$ in Step 2.
(4) Estimate the displacement with the coherence profile by Equation (2).

In the proposed method, the average of the coherence values is used to distinguish the height in the EV spectrum for the displacement estimation. Essentially, the selected height is the most scattering point in the height direction, and the noise in terms of the coherence becomes the least. With regards to the velocity range, the velocity center is set to 0 mm/year, then the velocity range is set from the negative half of the velocity ambiguity to the positive half of that. The coherence pattern is repeated with one range of the velocity ambiguity, so that it is critical to set and utilize the full range of the velocity spectrum for the displacement estimation.

## 4. Simulations and Verification

In this section, the simulation methods, conditions, and results are described in the following subsections, and the verification results of the proposed method are explained.

### 4.1. Simulation Method

In the simulation, NN-PSI including the proposed method was applied with the given different input displacements and observation conditions. Also, to confirm the difference between NN-PSI and ConvPSI, the displacement estimations of ConvPSI were also conducted, as shown in Figure 3. The influence of the observation interval was also evaluated by the simulation. Each of the simulation methods and results are explained in the following subsections.

#### 4.1.1. Displacement Types

There are many types of non-linear displacements, but in this experiment three types of displacements, step, exponential, and sinusoidal functions were used to simulate as non-linear displacements. The displacements by the step function can reproduce strong displacements in a very short period, these simulate the accelerated subsidence through an exponential function, and these simulate periodical displacements through the sinusoidal function. The conditions of the input displacement for simulation is summarized in Table 1. The amount of the displacement is expressed in the wavelength of sensor, λ, and each of the displacement type is explained as follow.

##### Step Displacement

The purpose of this simulation with the step function is to understand how the NN-PSI approach behaves with strong displacement in one interval of the observation. In the simulation, the amount of the displacement shown in Table 1 occurred in the middle of the given period. For the interferometric stacking technique, the limit of π on the differential phases corresponds to a maximum displacement of λ/4 over the revisit interval. Thus, in this simulation, the amount of the displacement occurred in one interval of the observation, a quarter of the wavelength (−0.25λ), was used.

##### Exponential Displacement

Using the exponential function for the input displacement in the simulation, it is possible to investigate if the NN-PSI enables to reconstruct the displacement with some accelerations. The input displacement phase was defined by the exponential functions, and about the half of the wavelength, 0.5λ. The displacement period was about 100 days, and in the rest of the period, the displacement was almost flat.

##### Sinusoidal Displacement

One of the most difficult displacements to be estimated with PSI approaches is a periodical displacement because most of the PSI is implemented with the linear model. The periodical displacement was defined with a sinusoidal function. The amount of the displacement was defined peak to peak, and this value was about the half of the sensor wavelength, 0.5λ, and the cycle of the displacement was set to twice in a year. The periodical displacement usually has one cycle a year; however, the additional cycle was added to this simulation to ensure that the NN-PSI was robust enough to reconstruct the sinusoidal displacements. In addition to the peak to peak displacement, the linear displacement of 0.25λ per year was also added to the input displacement for the simulation.

#### 4.1.2. Observation Conditions

The observation conditions used in the simulation are summarized in Table 2. In the table, the baseline distance is expressed as the baseline variance because the baseline distance for each interferometric pair was randomly selected with the range of the variance. Given the observation conditions, the height and velocity ambiguity were also calculated, and these values are described in the table. In the simulations, the height and velocity resolution was about 11 m and 11 mm/year, respectively, but the height and velocity step was set to 1 m and 1 mm/year to see the resulting EV spectrum in detail. 

#### 4.1.3. Observation Intervals

As introduced in Section 2.3, the observation intervals are the most important factor to decide the quality of the NN-PSI result. In this simulation, a sinusoidal function was selected because the widest velocity range in the EV spectrum is required in the estimation. The observation intervals and the amount of the peak to peak displacement were changed, so that the maximum displacement with the observation intervals can be estimated. 

The observation conditions were mostly followed in Table 2. However, to make this simulation more realistic than the previous conditions, the total observation period was set to 750 days, which is about two years. In order to change the observation intervals, the number of the observations was reduced from 76 through 61, so that the average of the observation interval could be changed. The total observation period was always kept 750 days in each simulation conditions, and each observation interval was randomly set 10 or 20 days according to the number of observation times. The change of the observation interval is directly reflected to the velocity ambiguity, according to Equation (4). The amount of the input sinusoidal displacement was changed from 0 through one λ, and the cycle of the sinusoidal function was also changed from two to one in a year. The calculation step was 0.1λ.

We used the root mean square errors (RMSE) between the input displacement and the NN-PSI results as an index of the evaluation, in order to understand the robustness with the sparse and irregular observation intervals in the NN-PSI estimations. Then it is possible to understand how the observation intervals impact the resulting displacement by NN-PSI, according to the RMSE.

### 4.2. Simulation Results

#### 4.2.1. Step Displacement

**Figure 5:**
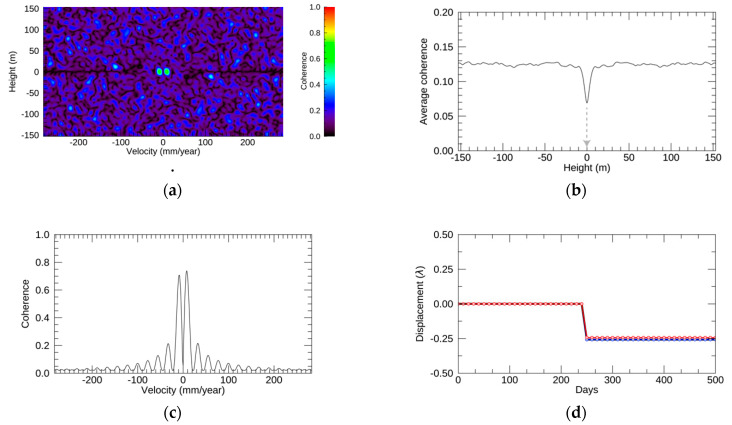
The simulation results with the step function. (**a**–**d**) The simulation outputs: EV spectrum, profile of the average coherence at each height along the velocity direction, profile of the coherence at the selected height along the velocity, and the original displacement in black and the estimated displacement by non-linear non-parametric persistent scatterer interferometry (NN-PSI) and conventional PSI (ConvPSI) in red and blue, respectively. The gray line in (**b**) indicates the selected height.

Regarding the simulation with step function, the resulting EV spectrum, profiles, and displacements are depicted in Figure 5. The EV spectrum in Figure 5a shows two clear lobes around 0 mm/year at the height of 0 m. Figure 5b shows the profiles of the average coherence along the velocity direction, and the height at the minimum average coherence was 0 m. Figure 5c shows the coherence profile at the selected height along the velocity direction, and two strong peaks were observed. These coherence values at the maximum were around 0.7. The resulting displacements by NN-PSI and ConvPSI are shown in Figure 5d, and the RMSE between the original displacement and those by PSI approaches was less than 0.007λ. It is clear that the step displacements with less than one quarter of the wavelength can be reconstructed by NN-PSI as well as by ConvPSI.

#### 4.2.2. Exponential Displacement

**Figure 6:**
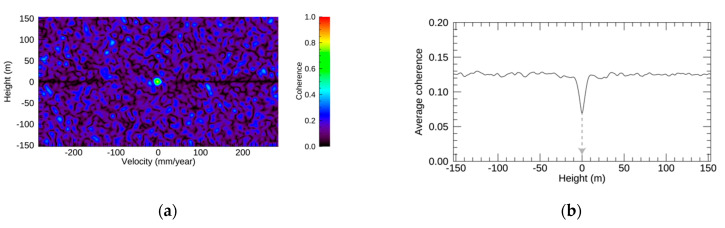

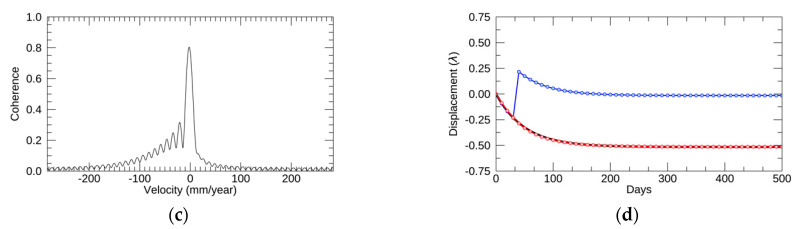
The simulation results with the exponential function. (**a**–**d**) The simulation outputs: EV spectrum, profile of the average coherence at each height along the velocity direction, profile of the coherence at the selected height along the velocity, and the original displacement in black, the estimated displacement by ConvPSI in blue, and NN-PSI in red, respectively. The gray line in (**b**) indicates the selected height.

Regarding the exponential function in the simulation, the resulting EV spectrum, profiles, and displacements are depicted in Figure 6. The EV spectrum in Figure 6a shows a very strong lobe around the center. Figure 6b shows that the height value at the minimum coherence value was 0 m. In Figure 6c, the profile along the velocity direction at the selected height shows a strong peak around the center of the velocity range. There were also several peaks with high coherence values in the negative velocity range in the figure, whereas there were no peaks with high coherence values in the positive velocity range. The RMSE between the original displacement and that by NN-PSI and ConvPSI were 0.006λ and 0.4λ, respectively. According to Figure 6d and the RMSEs, the estimation by ConvPSI had a jump because of the displacement over 2π ambiguity, whereas the reconstruction by NN-PSI fits with the original displacement very well.

#### 4.2.3. Sinusoidal Displacement

**Figure 7:**
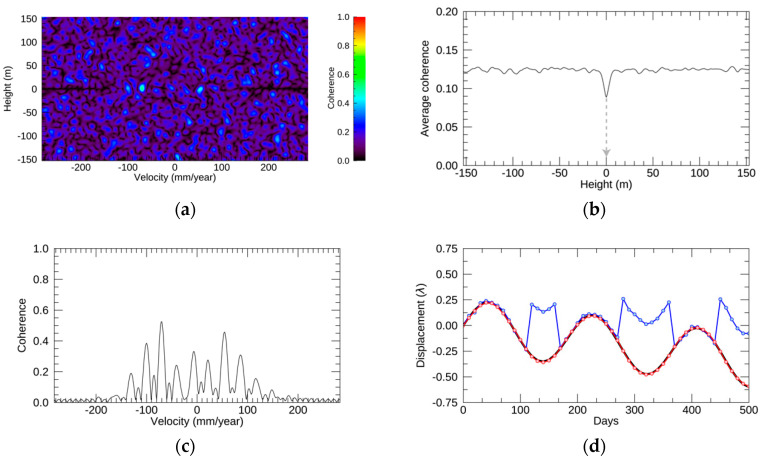
The simulation results with the sinusoidal function. (**a**–**d**) The simulation outputs: EV spectrum, profile of the average coherence at each height along the velocity direction, profile of the coherence at the selected height along the velocity, and the original displacement in black, the estimated displacement by ConvPSI in blue, and NN-PSI in red. The gray line in (**b**) indicates the selected height.

Regarding the sinusoidal function in the simulation, the resulting EV spectrum, profiles, and displacements are depicted in Figure 7. The EV spectrum in Figure 7a shows that the distribution of the strong coherence is not as clear as those of the step and exponential functions. Figure 7b shows that the height value at the minimum average coherence was 0 m. In Figure 7c, the coherence profile along the velocity direction shows many peaks, and the peaks are spread wider than those of the step and exponential functions. The profile indicates that the detection of the height with periodical displacements was difficult due to the many strong peaks that exist in the EV spectrum. According to Figure 7d, the estimation from NN-PSI fit with the original displacement, but the estimation of ConvPSI had several jumps. The RMSE values between the original displacement and that of NN-PSI and ConvPSI were 0.005λ and 0.2λ, respectively. NN-PSI was able to estimate certain periodical displacements that ConvPSI was unable to reconstruct.

#### 4.2.4. Observation Interval

**Figure 8:**
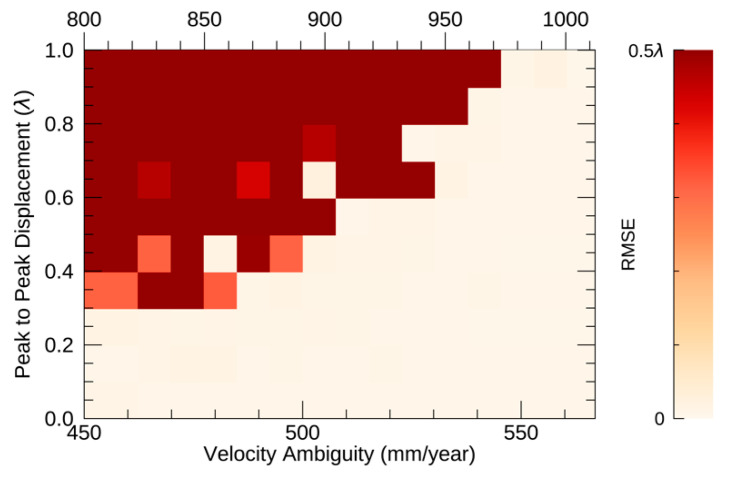
The vertical axis shows the amount of the displacement divided by the wavelength. The horizontal axes on the bottom and top show the velocity ambiguity of the X-band and C-band, respectively.

The simulation results of the observation interval are depicted in Figure 8. The range of the velocity ambiguity is changed by the wavelength of the sensor. Both X and C-band results are depicted in the figure. 

The white area in Figure 8 indicates that the estimation was correctly conducted by NN-PSI with an RMSE of less than 0.01λ. The range of the velocity ambiguity under the given condition was 450 to 566 mm/year for the X-band and 800 to 1012 mm/year for the C-band. Then, the minimum value of the velocity ambiguity with the displacement of 0.5λ was about 500 and 900 mm/year for the X-band and C-band, respectively. This result shows that the observation could be skipped nine times in 750 days to reconstruct the periodical displacement with a value of 0.5λ, and the required average observation interval was about 11 days.

As a result, the larger the amount of the displacement, the larger the velocity ambiguity is needed. Ideally, the observation should be regularly conducted in a short period for NN-PSI to obtain the correct estimation for large scale displacements that are equivalent to the sensor’s wavelength. Considering that this evaluation was conducted with the periodical displacement that requires the widest velocity ranges, the conditions for the other types of non-linear displacements can be mitigated. In summary, the observation interval and total observation period are important conditions to be considered for NN-PSI, and the required observation interval should be confirmed before the estimation of NN-PSI is conducted.

## 5. Real Data Processing

### 5.1. Study Area

**Figure 9:**
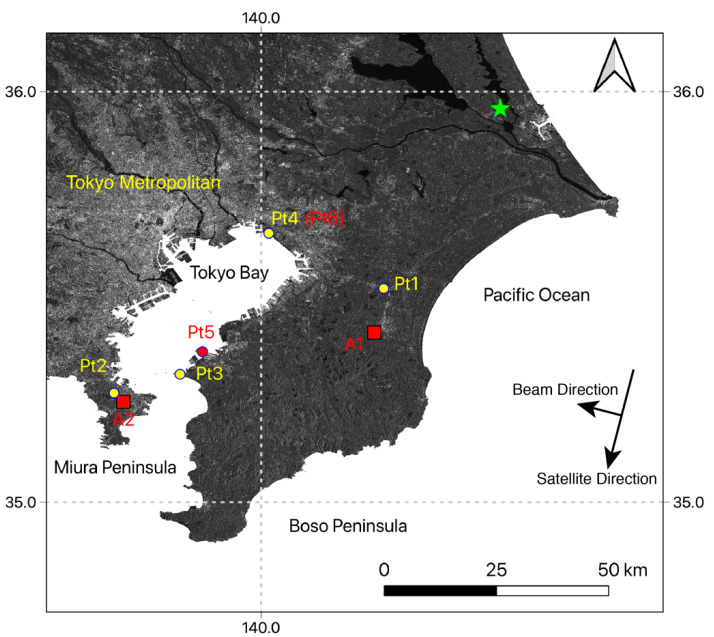
The average intensity image of Sentinel-1 in the study area. Pt1, Pt2, Pt3, and Pt4 are the location of the GNSS Earth Observation Network System (GEONET) stations. A1 and A2 are the evaluation areas, and Pt5 and Pt6 are the evaluation points. The green star is the location of the reference point for NN-PSI and ConvPSI.

The simulation results shown in the previous sections indicated that several types of non-linear displacements could be estimated with the proposed method. In this section, the validation of the simulation results was conducted with Sentinel-1 data that was acquired from the beginning of January in 2017 to the end of 2018. To see the displacements over a 2π ambiguity, the study area was set large, at roughly 25,000 km^2^, so that the difference between the reference displacement and the measurement displacements would be large enough. The study area is shown in Figure 9.

### 5.2. Method and Materials

**Figure 10:**
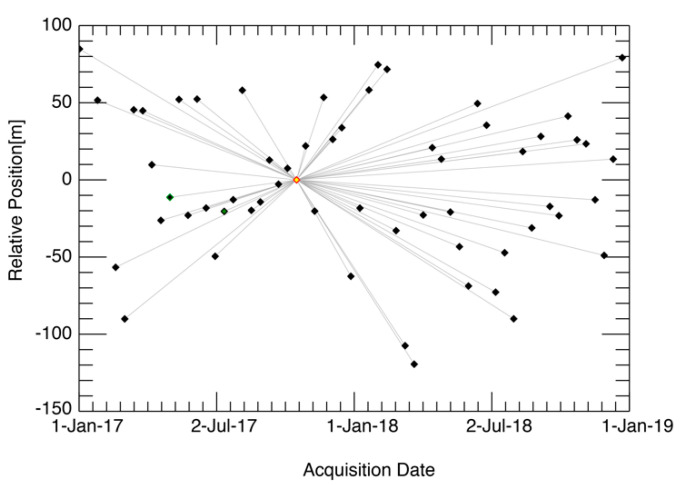
The combination of the interferometric pairs used in the PSI approaches. The yellow square shows the master acquisition, and the black squares are the slave acquisitions. The horizontal axis shows the date of the acquisition, the vertical axis shows the baseline length in meters, and the gray lines show the combination of the interferometric pairs.

The data description and the parameters used in NN-PSI are summarized in Table 3, and the distribution of the baseline distances is depicted in Figure 10. The baselines for Sentinel-1 is controlled within 50 m [28], and the height resolution was more than 100 m with the Sentinel-1 dataset. The objective of this experiment was not to distinguish the multiple scatterers in some tall buildings, and the large height resolution should not affect this experiment.

NN-PSI with the proposed methods was applied to Sentinel-1 data as shown in Figure 3. First, the estimated displacements by the NN-PSI approach were compared with the measured displacements at the GNSS Earth Observation Network System (GEONET) stations to confirm how the proposed method estimates the displacement with some ground truth data. Then, the reconstructed displacement close by the Pt1 and Pt2 shown in Figure 9 are investigated and compared with the displacement measured at GEONET. The RMSE between the NN-PSI and GEONET displacement was calculated for the validation. 

In this study area, some clear periodical displacements were observed by NN-PSI at Pt5 and Pt 6 in Figure 9. The observed displacements were compared with the displacement measured at the GEONET stations, Pt3 and Pt4 and the temperature measured at the closest meteorological stations operated by the Japan Metrological Agency (JMA). In this experiment, the NN-PSI results are also compared with those of ConvPSI.

### 5.3. Results of the Validation with GEONET

**Figure 11:**
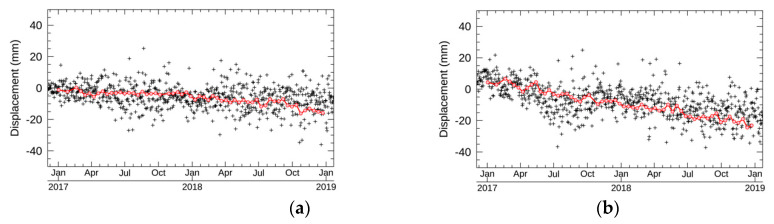
The black dot shows the GEONET displacements, and the red line shows the displacements estimated by NN-PSI. (**a**,**b**) the displacements at Pt1 and Pt2, respectively.

The validated displacements estimated by NN-PSI were the closest to the stations, Pt1 and Pt2, with high temporal coherence values around or higher than 0.7. The resulting displacements are shown in Figure 11. The RMSEs are summarized in Table 4.

According to Figure 11, the estimated displacements at Pt1 and Pt2 by NN-PSI correspond to those of GEONET. The comparison between NN-PSI and GEONET was conducted by averaging the GEONET displacement in two weeks, and the RMSEs were 2 and 3 mm at Pt1 and Pt2, respectively. Pt1 and Pt2 are located in the different peninsulas, and the distance of these points is about 75 km. This indicates that the displacements in a wide area can be estimated using NN-PSI. With the comparison result, we also confirmed that the estimation from NN-PSI was quite comparable to the GEONET measurement.

### 5.4. Periodical Displacement

**Figure 12:**
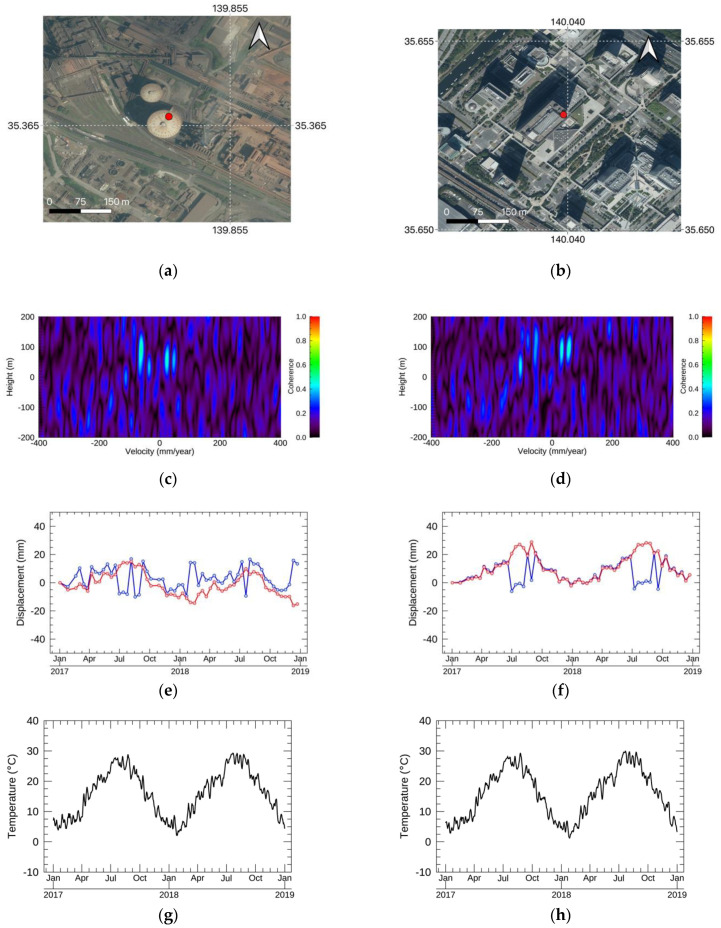

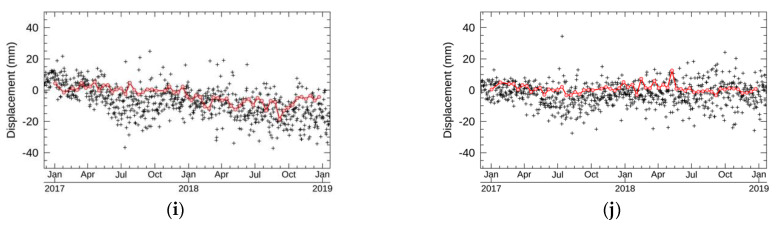
(**a**,**c**,**e**) The location of the displacement, EV spectrum, and NN-PSI in red and ConvPSI in blue at Pt5. (**b**,**d**,**f**) The location of the displacement, EV spectrum, NN-PSI in red, and ConvPSI in blue at Pt6. (**g**,**h**) The daily average temperature measured by the Japan Metrological Agency at the closest stations from Pt5 and Pt6. (**i**,**j**) GEONET measurement in black and NN-PSI in red at Pt3 and Pt4. The satellite imagery in (**a**,**b**) is distributed in the GSImap [29].

The periodical displacements were observed on a blast furnace in an iron mill at Pt5 and on the top of the large building at Pt6. The locations, the NN-PSI results, and other information are illustrated in Figure 12.

The EV spectrum in Figure 12c,d indicates that the residual height was 58 m and 91 m, respectively. Considering that the reflection is from the top of the tall buildings, these height values are reasonable. Since the type of the displacement is periodical at Pt5 and Pt6, several strong lobes were observed in the EV spectrum, and this pattern was also confirmed in the simulation shown in Figure 7a. The displacements estimated by NN-PSI, shown in Figure 12e,f have a one-year cycle, and the displacement moved toward the sensor direction in August, which is summer in Japan, and moved away from the sensor in February.

## 6. Discussion

### 6.1. Evaluation of the Proposed Method

According to the results obtained in the simulation, all kinds of displacements can be reconstructed with the proposed method. Using the full velocity range defined by the height and velocity ambiguities for the EV spectrum and the minimum of the average coherence along the velocity direction in EV spectrum, the height for the displacement estimation can be detected.

For the periodical displacement, the distribution of the strong lobes in EV spectrum is quite complicated, and it is clear that the bottom of the coherence profile gets shallower in estimating the sinusoidal displacement than others. Although it makes harder to detect the height for the displacement estimation, the proposed method works properly in the simulation as shown in Figure 5b, Figure 6b and Figure 7b. 

The feasibility of the NN-PSI approach depends on the observation frequency of the data rather than the displacement types. As shown in Figure 8, the velocity ambiguity should be large enough; that is, the observation interval should be small. Currently the most frequent and stable observation has been conducted by Sentinel-1, and the periodical displacements of about 2 cm of peak to peak can be estimated with this dataset by NN-PSI. In the future, more constellation SAR satellites are expected to launch; thus, the limitation of the observation frequency will be mitigated, and there should be more opportunities to have available datasets to apply for NN-PSI.

### 6.2. Periodical Displacements

The peak-to-peak displacements at both Pt5 and Pt6 were about 2–3 cm, and these values are close to the simulation result of the observation intervals. The estimation by ConvPSI had several jumps due to the sinusoidal displacement. The temperature trends are shown in Figure 12g,h, and the displacements estimated by NN-PSI follow the trends. Figure 12i,j show the displacements at the closest GEONET station from Pt5 and Pt6, and both the GEONET measurements and NN-PSI estimations did not show any periodical trends. This also indicates that the periodical displacements estimated by NN-PSI were independent from the atmospheric disturbance. Thus, the displacements shown in Figure 12e,f are the deformation influenced by the thermal expansion of the buildings. Quantitative validations regarding the sinusoidal displacements are still required; however, these results indicate that NN-PSI was capable of correctly estimating the periodical displacements that ConvPSI wrongly estimated.

Non-linear displacements in general are treated as a local displacement, and the local model cannot be used for the other regions of the interests. Usually, it is necessary to build some displacement models to reconstruct the periodical displacements as shown in [18]. As Figure 12 shows, the periodical displacements in two different sites were reconstructed reasonably by NN-PSI without any tunings. In addition to the periodical displacements, the linear and other types of the displacements can be reconstructed correctly by NN-PSI according to the simulations and real data processing. This means that NN-PSI is completely independent from the displacement models.

### 6.3. Other Improvement by NN-PSI

**Figure 13:**
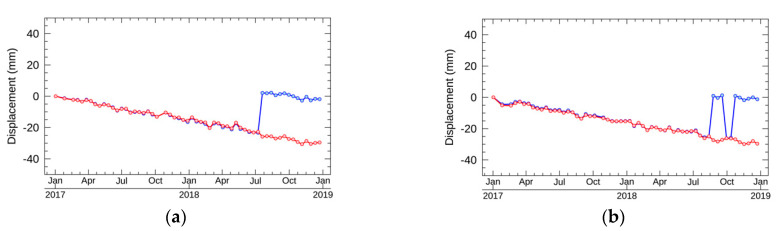
The red and blue lines show the resulting displacement of NN-PSI and ConvPSI, respectively. (**a**) The representative displacement in A1, and (**b**) in A2.

The improvement by NN-PSI was also investigated with Sentinel-1 and compared with the resulting displacement by ConvPSI, whose estimation is limited to the linear model. About a hundred investigation points with high mu and sigma of the backscatter coefficient were randomly selected in A1 and A2, shown in Figure 9. With the non-linear displacement, the temporal coherence, which indicates the fitting quality of the linear model [2,17], was not reliable, and thus the backscatter mu and sigma were used as an index of the stable phase. The representative time evolutions of NN-PSI and ConvPSI are shown in Figure 13.

According to Figure 13, the estimated displacements show the subsidence trend. There are clear phase jumps in the estimated displacements by ConvPSI, whereas those of NN-PSI do not show any jumps and continue the subsidence trend. These evaluation areas are close to the GEONET stations, and each of the distance from the GEONET station is less than 10 km. There are no jumps with the displacements of GEONET. Thus, the jumps in ConvPSI are likely caused by the displacement over the 2π ambiguity because the distances between A1 and A2 and the reference point are long enough to make the phase difference large.

In addition to the investigation of the time evolutions, the mean velocity of all investigated points was calculated to quantitatively confirm the displacements. Since the jumps in the ConvPSI displacements were mostly observed in the second half of 2018, the mean velocity of NN-PSI and ConvPSI between January to June in 2018 (T1) and between July to December in 2018 (T2) were evaluated. The results are summarized in Table 5, and the average of the mean velocity by ConvPSI in T2 was positive while that of NN-PSI in T2 was negative in both A1 and A2. This indicates that the displacement estimation by ConvPSI was not correctly calculated due to the limitation of the 2π displacement ambiguity. On the other hand, the NN-PSI technique should estimate the displacements correctly in the entire area.

**Figure 14:**
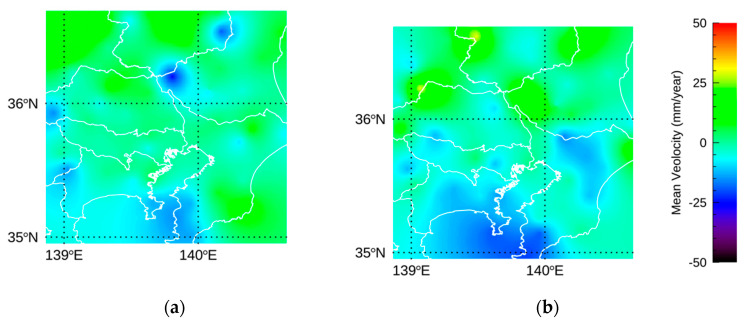
(**a**) The mean velocity calculated with GEONET data of T1, and (**b**) that of T2. The white lines show the boundaries of the prefectures in Japan.

In this study area and the period of the total observations, a slow slip event (SSE) was reported in the middle of 2018 by the Geospatial Information Authority of Japan [30]. The mean velocity of T1 and T2 in the study area was calculated with GEONET data using a Kriging interpolation [31] as shown in Figure 14. The amount of the mean velocity in this figure was converted to the line of sight of Sentinel-1 data. The amount of the subsidence in the Boso and Miura Peninsula was larger in the second half of 2018 due to the SSE. The period of the jump in the ConvPSI estimation also corresponded to the period of the SSE. We assumed that the displacements induced by the SSE influenced the amount of the displacement around A1 and A2, and the displacements were wrongly estimated by ConvPSI.

These results clearly show the limitation of the ConvPSI approach as well as the improvement by NN-PSI. The phase difference between the measurement and reference point gets bigger when the distance between these points gets longer, and the distance is more than 50 km. The phase difference became over 2π ambiguity due to the distance, and ConvPSI needs to use spatial or temporal unwrapping in order to conduct the correct displacement estimation. However, the displacement with 2π ambiguity can be reconstructed by NN-PSI without any spatial unwrapping. This is the significant improvement because the estimation can be done with the complete single scattering point. It also makes it available to reconstruct large scale displacements, such as earthquakes, landslides, and other types displacements.

### 6.4. Atomospheric Correction

Another consideration about NN-PSI is the atmospheric correction. In the experiment with Sentinel-1 data, the standard atmospheric correction [2] that is based on the linear displacement model was applied in both NN-PSI and ConvPSI. Basically, the standard atmospheric correction removes the phase that does not fit to the linear model, and there is some possibility that the non-linear displacement phase might be removed as noise. To improve the accuracy of the NN-PSI estimation, a new atmospheric correction designed for NN-PSI should be implemented in the future.

## 7. Conclusions

In this paper, we have proposed optimized parameters and calculation procedures for NN-PSI and verified these with simulations. The proposed method was also validated with the real data processing of the Sentinel-1 dataset.

The simulation results indicate that the step, exponential, and sinusoidal displacements can be estimated correctly without using any empirical parameters and models. The velocity range can be decided by the full range of the velocity ambiguity of the input dataset, and the precise residual height can be detected using the average coherence of each height in the EV spectrum. With the experiment by the simulations, the calculation procedure in NN-PSI was verified, and these outputs ensure that the displacement estimation by NN-PSI is stable and robust with several types of non-linear displacements.

With real data processing, we confirmed that NN-PSI is also capable of estimating several types of displacement in the wide area. The accuracy of the resulting displacements was comparable to the GEONET measurements, and the RMSE was less than 3 mm in two years. The displacements that exceeded 2π displacement ambiguity as well as periodical displacements were correctly estimated by the proposed method in NN-PSI.

In conclusion, the NN-PSI approach is an extended PSI approach designed for the non-linear displacement. This approach can utilize the maximum spatial resolutions and phase information because the spatial filters, spatial phase unwrapping, and displacement models are not implemented. We confirmed that NN-PSI can overcome the main drawbacks of the conventional PSI approaches on certain linear models, and this proposed method is a breakthrough for displacement monitoring using interferometric stacking techniques.

## Figures and Tables

**Table 1:** The simulation conditions for the non-linear displacement.

Displacement Type	Total Amount of the Simulated Displacement in 500 Days
Step	−0.25λ
Exponential	−0.5λ
Sinusoidal ^1^	0.5λ

^1^ The amount of the displacement indicates the peak to peak value.

**Table 2:** The observation conditions for the elevation-velocity (EV) spectrum.

Items	Values
Slant range distance	700 km
Wavelength	1 λ
Incidence angle	45°
Baseline variance ^1^	±6%
Backscatter coefficient	5 dB
Number of observations	51
Observation interval	10 days
Total observation period	500 days
Simulated height	0 m
Height ambiguity	306 m
Height step	1 m
Velocity ambiguity	566 mm/year
Velocity step	1 mm/year

^1^ The value in this column is the ratio of the baseline and the critical baseline.

**Table 3:** Data description.

Parameters	Values
Satellite sensor	Sentinel-1
Monitoring period	January 2017–December 2018
Number of acquisitions	59
Time interval of the acquisitions	12 days
Date of the master acquisition	16 October 2017
Incidence angle	39.0°
Wavelength (λ)	55.5 mm
Average baseline distance	39.1 m
Average temporal baseline	12.6 days
Height resolution	113.8 m
Height ambiguity	585.6 m
Velocity resolution	14.1 mm/year
Velocity ambiguity	802.1 mm/year

**Table 4:** The RMSEs between the NN-PSI estimated displacement and GEONET measurements.

Scheme	RMSE (mm)	Temporal Coherence
Pt1	2.1	0.69
Pt2	3.5	0.77

**Table 5:** The average velocity estimated by the PSI approaches.

Method	T1 (mm/year)	T2 (mm/year)
**A1**		
NN-PSI	−17.1	−19.0
ConvPSI	−10.0	28.8
**A2**		
NN-PSI	−14.1	−17.9
ConvPSI	−12.8	49.4

## Data Availability

Not applicable.
